# The evaluation of shotgun sequencing and *rpoB* metabarcoding for taxonomic profiling of bacterial communities

**DOI:** 10.1186/s12866-025-04149-3

**Published:** 2025-07-04

**Authors:** Karine Durand, Jean-Claude Ogier, Kiwoong Nam

**Affiliations:** DGIMI, INRAE, Univ Montpellier, Montpellier, France

**Keywords:** Metabarcoding, Metagenomics, Microbial profiling, *rpoB* metabarcoding, Whole-genome shotgun sequencing

## Abstract

**Background:**

The importance of microbial community profiling has been increasingly recognized in biological and environmental research. While metabarcoding has been widely used for such analysis by targeting specific DNA sequences as markers, shotgun sequencing has been proposed as an alternative method because the analysis of whole genomes can potentially reduce biases introduced by targeted approaches. However, it is largely unknown whether shotgun sequencing may provide improved precision for qualitative taxonomic identification and quantitative abundance estimation compared with metabarcoding with housekeeping gene markers, such as the *rpoB* gene. Furthermore, the comparative performance of various bioinformatics pipelines for shotgun data analysis remains uncertain. In this study, we evaluated the performance of *rpoB* metabarcoding and shotgun sequencing coupled to various bioinformatic pipelines to describe the bacterial diversity of artificially generated mock bacterial communities, which included eukaryote gDNA intentional contamination or whole-genome amplification. For shotgun sequencing, the Assembly-Binning-Method and k-mer-based approaches were evaluated.

**Results:**

For taxonomic profiling, the Assembly-Binning-Method and *rpoB* metabarcoding exhibited comparable sensitivity and precision, whereas k-mer approaches produced a notably high number of false negatives. In some cases, the Assembly-Binning-Method improved taxonomic resolution compared with *rpoB* metabarcoding by identifying taxa at the species level rather than the genus level. Regarding the quantification of microbial composition, the Assembly-Binning-Method consistently showed a higher correlation with expected values ​​and a lower dissimilarity index than *rpoB* metabarcoding. The use of three sets of reference genomes to calculate depth coverage did not systematically affect the precision of the Assembly-Binning-Method.

**Conclusions:**

These results demonstrate that although shotgun sequencing and *rpoB* metabarcoding have nearly equivalent accuracy in taxonomic profiling, shotgun sequencing has better taxonomic resolution and outperforms *rpoB* metabarcoding in quantitative estimation of microbial community abundance using the Assembly-Binning approach.

**Supplementary Information:**

The online version contains supplementary material available at 10.1186/s12866-025-04149-3.

## Background

Analyzing microbial communities is one of the important steps in understanding ecosystem dynamics, human health, and biotechnology, as the role of microbiota has been increasingly recognized [1, 2]. This type of analysis typically includes the identification of microbial composition and the detection of microbial shifts in response to various environmental conditions, such as climate change or host-microbe interactions. Metabarcoding has been widely used for this purpose by sequencing a specific molecular marker [3]. Taxonomic composition is determined by comparing these sequences against a reference database. The 16S rRNA gene has long been used as a reference marker with the support of a large database for bacterial profiling. As an alternative, single-copy genes such as *gyrB* and *rpoB* have also been suggested as a marker to improve the taxonomic profiling [4, 5]. *rpoB* metabarcoding, for example, was shown to identify microbial communities with a resolution up to the species level in infective juvenile nematodes [6]. However, potential variabilities of primer efficiency among species might lead to differential rates of DNA amplification [7, 8], possibly leading to biased quantitative estimation of the abundance [9, 10]. Therefore, metabarcoding needs to be used cautiously for the quantitative analysis [6].

Shotgun sequencing can overcome these limitations using whole genome sequences within a microbial community without targeting any markers. In this method, the genomic DNA from whole organisms in a sample is randomly fragmented, and high-throughput sequencing is subsequently performed [11]. Reads are then processed using bioinformatics tools to assign taxonomies, estimate relative abundances, or reconstruct genomes. Shotgun sequencing can also provide additional insights by identifying functional traits within complex communities. The usage of shotgun sequencing has been further prompted by a reduction in the cost of sequencing [12].

Bioinformatics tools for taxonomic assignment and quantification of metagenomic shotgun sequencing data can be classified into two groups. The first group is based on the k-mer approach. Kraken2 software [13], for example, breaks reads into smaller k-mers and compares them to a database of reference k-mers generated from genomic sequences with known species identity. For each query k-mer, Kraken2 identifies all matches in the database and searches for their closest common ancestor in the taxonomic tree. Bracken [14] uses the output of Kraken2 and probabilistically re-distributes the reads in the taxonomic tree to refine abundance estimates. Sourmash [15] is another k-mer-based tool using MinHash sketches, which are compact subsets of k-mers, for increased efficiency.

The other group is the Assembly-Binning-Method. This method involves de novo genome assembly into contigs out of sequencing reads. Subsequently, the reads are mapped against these contigs to calculate the read depth and the tetranucleotide frequency, enabling the clustering of similar contigs into metagenome-assembled genomes (MAGs). The taxonomic assignment is performed using a comparison with reference databases. For example, skani [16] uses the average nucleotide identity (ANI) and the aligned fraction to determine the taxonomic identity of MAGs. The relative abundance of taxa is estimated using the depth-of-coverage approach by counting the number of reads mapped to MAGs or reference genomes.

Previous studies demonstrated that shotgun sequencing provides a higher resolution of taxonomic identification to the species level with more sensitive detection of rare taxa than 16S rRNA metabarcoding [17–19]. The *rpoB* marker was demonstrated to provide improved taxonomic resolution compared with 16S rRNA by identifying taxa up to the species level, but it is still unclear if shotgun sequencing could outperform *rpoB* metabarcoding. Furthermore, between k-mer approach and Assembly-Binning-Method, it is largely unknown which method provides more precise taxonomic profiling and quantitative abundance estimation.

In this study, we compared the performance between shotgun sequencing using various bioinformatic tools and *rpoB* metabarcoding in terms of qualitative precision and taxonomic resolution to identify species composition and quantification precision to estimate the relative abundance. We used artificially generated mock bacterial communities with varying microbial compositions of nineteen taxa [6]. We also generated a mock community intentionally contaminated by *Spodoptera frugiperda* to mimic natural experimental conditions. Furthermore, an amplification step was added to three mocks during shotgun sequencing to test its effect on abundance estimation.

## Materials and methods

### Mock communities preparation

Nineteen bacterial isolates encompassing a broad taxonomic diversity among eubacteria (16 Pseudomonadota, two Bacillota, and one Bacteroidota) were selected to compose the same five reference mock communities as Ogier et al. [6] (Table 1). In addition, to replicate the potential contamination that might occur during DNA extraction within their natural host, gDNA from *S. frugiperda* was added to the artificial mock community mock5 to create mock5M.

**Table 1:** The mock communities used in this study with the list of taxa and their relative proportions (%)

Isolates	Mock1	Mock2	Mock3	Mock4	Mock5
*Brevundimonas diminuta*	6.483	0.419	10.985	11.366	11.410
*Alcaligenes faecalis*	1.663	0.107	2.818	2.916	2.927
*Ochrobactrum anthropi*	2.269	0.147	3.845	3.978	3.994
*Serratia liquefaciens*	1.528	0.099	2.589	2.678	2.689
*Photorhabdus luminescens*	5.398	59.639	0.401	0.040	0.005
*Stenotrophomonas maltophilia*	8.848	0.572	14.993	15.512	15.573
*Xenorhabdus nematophila*	37.810	36.691	3.367	0.395	0.040
*Variovorax paradoxus*	2.416	0.156	4.093	4.235	4.252
*Delftia acidovorans*	1.491	0.096	2.527	2.614	2.624
*Enterococcus mundtii*	0.099	0.006	0.167	0.173	0.174
*Pseudomonas chlororaphis*	3.261	0.211	5.526	5.718	5.740
*Pseudomonas protegens*	2.187	0.141	3.706	3.835	3.850
*Pseudomonas putida*	3.278	0.212	5.555	5.747	5.770
*Acidovorax sp.*	2.359	0.152	3.998	4.136	4.152
*Acinetobacter sp.*	6.548	0.423	11.095	11.480	11.525
*Paenibacillus sp.*	3.385	0.219	5.736	5.935	5.958
*Sphingobacterium sp.*	6.024	0.389	10.207	10.560	10.602
*Sphingomonas sp.*	3.453	0.223	5.851	6.054	6.077
*Achromobacter sp.*	1.500	0.097	2.541	2.629	2.639

The total gDNA quantity was 4278 ng in mock1, 523 ng in mock2, 923 ng in mock3, 1668 ng in mock4, and 133 ng in mock5. Mock5M was generated by adding 90 ng of *S. frugiperda* gDNA to mock5 to simulate natural sample conditions. In mock1, *Xenorhabdus nematophila* was the predominant species, exhibiting a high relative abundance (37.8%) (Table 1). In mock2, both *X. nematophila* (36.7%) and *Photorhabdus luminescens* (59.6%) were the two most abundant species, while other taxa were present at very low relative abundances (< 0.6%). The coefficients of variation for taxonomic compositions in mock1 and mock2 were 1.55 and 2.96, respectively, which were higher than those in mock3 (0.74), mock4 (0.80), and mock5 (0.81), meaning that mock3, mock4, and mock5 had more balanced abundance distributions of different taxa than mock1 and mock2.

### Data generation

#### RpoB sequencing

The *rpoB* regions were amplified following the protocol described by Ogier et al. (2019). Briefly, we used the previously designed primers Univ_*rpoB*_deg_F (5'—GGYTWYGAAGTNCGHGACGTDCA—3') and Univ_*rpoB*_deg_R (5'—TGACGYTGCATGTTBGMRCCCATMA—3'), resulting in the generation of *rpoB* amplicons with 435 bp in size. Thirty-five amplification cycles were performed in a Bio-Rad thermocycler with 1 to 50 ng of genomic DNA, using high-fidelity iProof™ DNA Polymerase (Bio-Rad), and annealing temperatures of 57 °C. The presence of contaminating DNA was assessed in each PCR run by including negative controls with sterile ultra-pure water as the template. Amplicon DNA quantities and sizes were systematically analyzed by agarose gel electrophoresis.

All Illumina-indexed *rpoB* amplicons were purified, multiplexed, and sequenced at the Genseq platform (University of Montpellier, France). The library was generated as follows. The amplicon was purified using magnetic beads (Clean PCR, Proteigene, France), followed by an additional round of PCR in a total volume of 18 µL (5 µL of products from the first round of PCR, 9 µL of Phusion® High-Fidelity PCR Master Mix, NEB, France, 2 µL of index adapter I5, 2 µL of index adapter I7). Cycling conditions were as follows: 95 °C for 3 min, then 10 cycles of 95 °C 30 s, 55 °C 30 s, 72 °C 30 s, then final elongation for 5 min at 72 °C. A set of 384 index pairs based on IDT's (Integrated DNA Technologies) unique dual index set was used to mark all samples. After purification with magnetic beads, these final PCR products were multiplexed. Paired-end sequencing with 300 bp read length was performed using an Illumina MiSeq sequencer using the MiSeq v3 reagent kit (600 cycles; Illumina) according to the manufacturer’s instructions. FastQ files were generated at the end of the run within the sequencer using bcl2fastq [20]. The quality of the raw data was assessed using a module developed by the Montpellier Bioinformatics Biodiversity platform with the MultiQC program [21, 22].

#### Shotgun sequencing

Libraries were generated using the TruSeq DNA PCR-Free kit for mock1, mock3, and mock4, and the TruSeq Nano DNA kit for mock2, mock5, and mock5M with the 350 bp insert size. Paired-end Illumina sequencing was performed using Novaseq S6000. FastQC-v0.12.1 [23] was used with the default parameters to assess the read quality. We used Trimmonatic 0.36 [24] to discard adapter sequences and low-quality bases within reads. To remove the *S. frugiperda* contaminant reads in the mock5M, we used Bowtie2 v2.3.4.1 [25] with -very-sensitive-local preset for mapping against the *S. frugiperda* reference genome ver7 [26] (https://bipaa.genouest.org/sp/spodoptera_frugiperda_pub/download). Samtools v1.9 [27] was used to extract the reads not mapped to the *S. frugiperda* reference genome for downstream analysis.

### Taxonomic assignment

To address the known heterogeneity of nomenclature between databases, we considered *Photorhabdus luminescens* and *Photorhabdus laumondii * [28], and *Brucella anthropi* and O*chrobactrum anthropi* [29] as synonyms. The *rpoB* sequences [6] were processed using the FROGS pipeline (Find Rapidly OTUs with Galaxy Solution) v4.1.0 [30]. Swarm v3.0.0 [21] was used for sequence clustering, and chimeric sequences were identified and removed using VSEARCH v2.17.0 [31] with the de novo UCHIME method. Clusters with read abundances below 0.005% were filtered out. The sequences were clustered into Amplicon Sequence Variants (ASVs), and taxonomic assignments were performed using the RDP Classifier v2.10.2 [32] by using the *rpoB* database (https://vm-galaxy-prod.toulouse.inrae.fr/galaxy_main).

We analyzed the shotgun sequencing using three k-mer methods: Kraken2 v2.1.2 [33] with Kraken2_Standard_set database, followed by Bracken [14] for Bayesian re-estimation of abundances with minikraken2_v2_8GB_201904_UPDATE database and Sourmash v4.8.6 [15], a k-mer-based tool using MinHash sketches with an LCA algorithm. Sourmash was used with two kmer sizes, 31 bp and 51 bp, and the NCBI database genbank-2022.03-bacteria-k31, and genbank-2022.03-bacteria-k51 downloaded from (https://sourmash.readthedocs.io/en/latest/databases.html). For Kraken2, Bracken, and Sourmash, we used a minimum detection threshold set at 1% of the total reads to limit errors and avoid excessive false positives or background noise.

For Assembly-Binning-Method, the reads from each mock community were assembled into contigs using metaSPAdes v3.15.3 [34]. The resulting scaffolds were then binned using MetaBAT2 v1.7 [35] to cluster the scaffolds into groups representing the whole genome of an organism. The completeness was estimated as the proportion of a collocated set of genes that are ubiquitous and single-copy present in a MAG compared to those present in a closely related reference genome, and the contamination of these MAGs was evaluated using CheckM v1.0.18 [36]. MAGs with a contamination rate higher than 5% were removed. These analyses were performed within the KBase platform using its integrated tools [37]. Skani v0.2.2 [16] was used for the taxonomic assignment of all MAGs, which calculates genome similarity with the average nucleotide identity (ANI), using the GTDB-Tk 2.4.0 database [38]. We considered only taxonomic assignment with ANI > 95% for taxa defined at the species level and > 80% for those at the genus level using the NCBI Taxonomy as the reference.

### Relative abundance estimation

For *rpoB* metabarcoding, the calculation of relative abundance is based on the ASV table generated by the FROGS pipeline (Table S1). For shotgun sequencing, the relative abundance was calculated based on the genomic average read obtained by mapping reads to reference genomes representing the taxa in the mock communities (CovDepth). We employed three sets of reference genomes in our analysis, referred to as CovDepth_MAG, CovDepth_NCBI, and CovDepth_Skani. First, we selected one representative MAG per taxon, choosing the one with the most complete genome as a reference genome for mapping. Second, we used NCBI reference genomes representing the mock taxa. When a taxon was identified at the genus level, we selected three different species within the genus to account for genetic diversity (Table S2). Third, we used the genomes identified by skani v0.2.2 from the Genome Taxonomy Database (GTDB-Tk 2.4.0) [38] as the closest relatives of the MAGs. Reads were aligned to these three sets of reference genomes using Bowtie2 v2.3.4.1 [25] with the–very-sensitive-local option, and read depth was obtained from the resulting bam files using Samtools v1.9 [27]. When three species from the NCBI reference genome were used, the read depth was averaged out.

### Statistical analysis

For *rpoB* metabarcoding, rarefaction curves were generated using the R packages of Phyloseq [39], Vegan v2.6-4 [40], and ranacapa [41] as previously described [6]. We evaluated the performance of all methods of taxonomic identification using precision (Precision = true positives/(true positives + false positives)), recall (Recall = true positives/(true positives + false negatives)), and F1-score (F1 Score = (2 × precision × recall)/(precision + recall)), as a harmonic mean of the precision and recall rate.

To compare the correctness of the methods, we calculated the deviation of estimated abundances from expected values using the Bray-Curtis dissimilarity index with the R package vegan v2.6-10. When an isolate was not detected, its abundance was assigned a value of 0 to ensure consistency across comparisons. Wilcoxon test and Spearman’s correlation test were performed using R [42].

## Result

### Metabarcoding and shotgun sequencing

The *rpoB* amplicons were sequenced using Illumina MiSeq technology. The number of reads per sample and the ASV composition are detailed in Table S1. *rpoB* metabarcoding yielded an average of 18,946 reads (rarefaction curves are shown in Figure S1). Shotgun sequencing yielded an average of 78.91 million reads and 11.83 Gbp per mock. In mock5M, after discarding the reads mapped to the *S. frugiperda* genome [26], 37.4 million out of 74.9 million reads remained. These filtered reads have 5.5 Gbp in total size. The Assembly-Binning-Method produced between 27 and 41 MAGs per mock. Completeness of the MAGs ranged from 0.16% to 100% (mean 48.2%). Only six out of the total of 194 MAGs (3.09%) were discarded due to the potential of mis-assemblies or mis-binning (*i.e.*, > 5% contamination rate, as suggested by the original method paper [35]), and the remaining 188 MAGs were used in subsequent analyses (Table S3).

### Taxonomic assignment

**Fig. 1:**
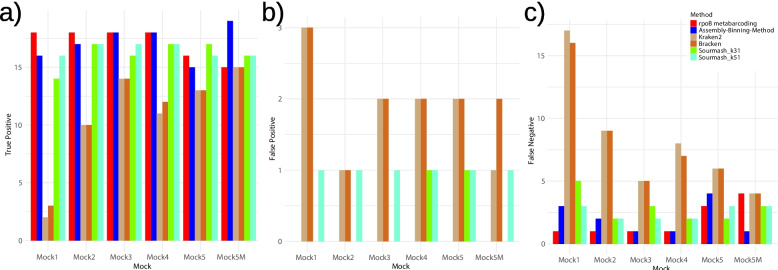
Bar plots showing the number of (**a)** true positives, (**b**) false positives, and (**c**) false negatives across mock communities for each method

In total, the *rpoB* metabarcoding identified 15 to 18 true positives out of the 19 taxa for each mock, with an average of 17.1 (Fig. 1a). Kraken2, Bracken, Sourmash-k31 (k-mer length = 31 bp), and Sourmash-k51 (k-mer length = 51 bp) identified between 2 and 15, 3 and 15, 14 and 17, and 16 and 17 true positives, with average values of 10.8, 11.2, 16.1, and 16.5, respectively. The Assembly-Binning-Method identified between 15 and 18 true positives with an average of 17.0 using skani [16].

No false positives were detected with the *rpoB* metabarcoding and Assembly-Binning-Method (Fig. 1b). On the other hand, k-mer methods generated false positives ranging from one to three, one to three, zero to one, and one, with averages of 1.83, two, 0.33, and one in Kraken2, Bracken, Sourmash-k31, and Sourmash-k51, respectively. The number of false negatives ranged from one to four for *rpoB* metabarcoding, with an average of 1.83 (Fig. 1c). In particular, *Sphingobacterium* was not detected in any mock communities (Table S4). For Assembly-Binning-Method, false negatives ranged from one to four, with an average of two. Particularly, the Assembly-Binning-Method reduced the number of false positives from four to one in mock5M compared to *rpoB* metabarcoding. Kraken2, Bracken, Sourmash-k31, and Sourmash-k51 identified false negatives ranging from 4 to 17, 4 to 16, 2 to 5, and 2 to 3, respectively, with averages of 8.16, 7.83, 2.83, and 2.5.

**Fig. 2:**
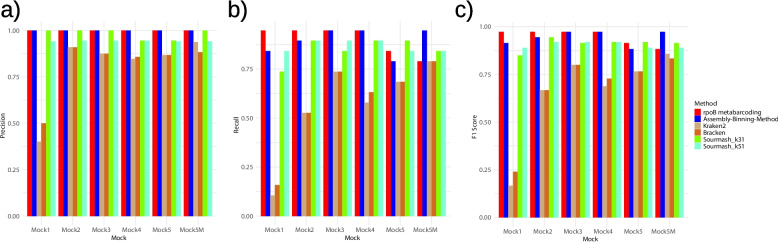
Barplots showing (**a**) the precision, (**b**) the recall rate, and (**c**) the F1-score for each mock community for each method

*rpoB* metabarcoding and Assembly-Binning-Method always have 100% of precision across the mocks, which was a significantly higher than Kraken2 (range: 0.4–0.938, mean = 0.81, two-tailed Wilcoxon test, *p* = 0.036), Bracken (range: 0.5-0.909, mean = 0.82, *p* = 0.036), and Sourmash_k51 (range: 0.941–0.944, mean = 0.94, *p* = 0.032), but not than Sourmash_k31 (range: 0.944-1, mean = 0.98, *p* = 0.3457) (Fig. 2a). The *rpoB* metabarcoding and the Assembly-Binning-Method showed comparable mean recall rates of 0.90 (range: 0.789-0.947) and 0.89 (range: 0.789-0.947), respectively (Fig. 2b). Assembly-Binning-Method had higher recall rates than Kraken2 (mean = 0.57, range: 0.105-0.789, *p* = 0.036), Bracken (mean = 0.59, range: 0.158-0.789, *p* = 0.036), Sourmash_k31 (mean = 0.85, range: 0.737-0.895, *p* = 0.206) and Sourmash_k51 (mean = 0.87 range: 0.842–0.895, *p* = 0.203).

The average F1 score calculated from the precision and the recall rate across the mocks was 0.95 for *rpoB* metabarcoding (range: 0.882-0.973) and 0.94 for the Assembly-Binning-Method (range: 0.882-0.973), implying that the F1 scores are comparable between these two (Fig. 2c). *rpoB* metabarcoding and Assembly-Binning-Method had higher F1 scores than Kraken2 (range: 0.167-0.857, mean = 0.66, *p* = 0.036), Bracken (range: 0.240-0.833, mean = 0.67, *p* = 0.036), Sourmash_k31 (mean = 0.91, range: 0.848-0.944, *p* > 0.05), and Sourmash_k51 (mean = 0.90, range: 0.889-0.919, *p* > 0.5).

We also compared the resolution of taxonomic identification. The Assembly-Binning-Method identified four species, including *Sphingobacterium detergens*, *Acidovorax kalamii*, S*phingomonas koreensis*, and *Acinetobacter dispersus*, across all mocks, which were identified only at the genus level when *rpoB* metabarcoding was used. Sourmash_k31 and Sourmash_k51 also improved the resolution of the taxonomic identification from genus to species levels across all mocks for three species, including *S. detergens*, *A. kalamii*, and *Paenibacillus lautus*, compared with the *rpoB* metabarcoding.

### Quantification of microbial community composition

**Fig. 3:**
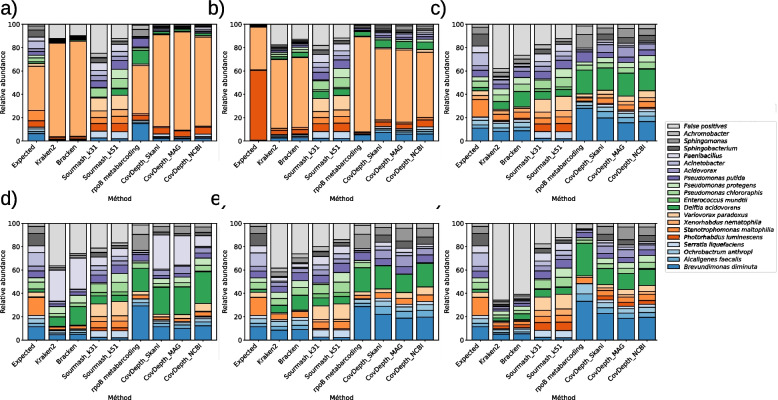
Composition bar plots of the relative abundances of taxa by methods compared to the expected abundance. **a**) mock1, **b**) mock2, **c**) mock3, **d**) mock4, **e**) mock5, and **f**) mock5M

**Fig. 4:**
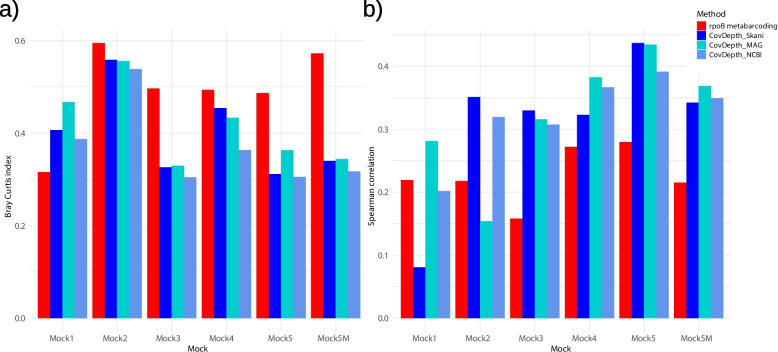
Comparison of quantification performance for each method across mock communities using **a**) Bray–Curtis dissimilarity index and **b**) Spearman correlation

To investigate the quantification of microbial composition, we focused only on the *rpoB* metabarcoding and Assembly-Binning-Method, as both demonstrated an absence of false positives, unlike k-mer methods with substantial rates of false positives (Figs. 1 and 3). ANI was highest for CovDepth_MAG, intermediate for CovDepth_Skani, and lowest for CovDepth_NCBI across all mock communities (Figure S2). We calculated the Bray-Curtis dissimilarity index to calculate the deviation of estimation from the expected taxa abundances. In all mocks except mock1, CovDepth methods yielded a lower Bray-Curtis dissimilarity index than *rpoB* metabarcoding (Fig. 4a). Among the CovDepth methods, CovDepth_NCBI showed the lowest mean Bray-Curtis dissimilarity index calculated from the mocks (with a mean of 0.384, range: 0.304-0.538), followed by CovDepth_Skani (0.411, range: 0.326-0.454), and CovDepth_MAG (0.430, range: 0.344-0.557). *RpoB* metabarcoding had an average Bray-Curtis dissimilarity index of 0.496 (range: 0.318-0.595). However, we did not observe a consistent trend among the CovDepth methods.

We also calculated Spearman’s correlation coefficient between the estimated and expected taxa abundances in each mock to compare the correctness of the estimated abundance ranks. Across all mocks, the highest correlation coefficient was observed from the CovDepth, rather than the *rpoB* metabarcoding (Fig. 4b). The correlation coefficients were highest for CovDepth_NCBI (with a mean of 0.354, range: 0.304-0.402), followed by CovDepth_Skani (0.348, range: 0.322-0.404) and CovDepth_MAG (0.343, range: 0.276-0.434), finally by *rpoB* metabarcoding (0.227, range: 0.158-0.279). Among the CovDepth methods, in mock4, mock5, and mock5M, CovDepth_MAG showed the highest Spearman correlation coefficient. CovDepth_NCBI had the highest correlation coefficient in mock1, and CovDepth_Skani showed the highest correlation coefficient in mock2 and mock3. This result implies, again, that no consistent trend is observed among the CovDepth methods.

We also compared the ability to classify taxa into two abundance classes: abundant (≥ 10%) and rare (< 10%). The average proportion of correct classifications was 0.825, ranging from 0.667 to 1, with no apparent differences among the methods (Figure S3). These results suggest that all methods can be reliably used for coarse abundance classification.

## Discussion

Shotgun sequencing has been proposed as an alternative to metabarcoding for qualitative and quantitative analysis of microbial communities. However, it remains largely unknown whether shotgun sequencing truly outperforms metabarcoding and which bioinformatics pipelines produce the most precise results among existing options. In this study, we performed a comparative analysis of methods based on shotgun sequencing and *rpoB* metabarcoding for taxonomic profiling and relative abundance estimation using six artificially generated bacterial communities.

For the qualitative analysis of taxonomic identification, Assembly-Binning-Method and *rpoB* metabarcoding had higher precision, recall rates, and F1 scores for taxonomic profiling than k-mer methods. While the difference between Assembly-Binning-Method and *rpoB* metabarcoding was not observed, Assembly-Binning-Method provided species-level resolution of four taxons. For example, *S. detergens* was identified only at the genus level across all mocks when *rpoB* metabarcoding was used. These results aligned with previous studies showing that shotgun sequencing improves the taxonomic identification of taxa that are poorly resolved by metabarcoding [43, 44]. Assembly-Binning-Method also outperformed *rpoB* metabarcoding in complex mock5M with intentional contamination of insect gDNA, by reducing the number of false negatives from 21.05% (4 out of 19) to 5.26% (1 out of 19), (Fig. 2), confirming previous findings that showed better performance of shotgun sequencing in samples containing host DNA [45]. Hence, Assembly-Binning-Method was shown to be the best option for correct taxonomic assignment in the analyzed mock communities. Unexpectedly, k-mer approaches resulted in rather high false positive rates, which may lead to misinterpretations of microbial community composition.

For the quantitative analysis to estimate the relative abundance of taxa within the mock communities, Assembly-Binning-Method with CovDepth methods generated better results than *rpoB* metabarcoding, while no differences were observed in the accuracy of coarse abundance classification. The CovDepth methods showed a lower Bray-Curtis dissimilarity index than *rpoB* metabarcoding in all mocks, implying that CovDepth methods show less deviation from true abundance (Fig. 4a). In addition, the CovDepth method had higher Spearman’s correlation coefficients than *rpoB* metabarcoding in all mocks, implying that the CovDepth methods provide more accurate taxonomic compositional ranks than *rpoB* metabarcoding (Fig. 4b). Notably, the three mocks with whole-genome amplification for next-generation sequencing library preparation did not exhibit significantly lower quantification precision than the rest mocks. These results suggest that, when gDNA quantities are insufficient for sequencing, whole-genome amplification can be used during library preparation without a major risk of introducing bias in abundance estimation. No method within CovDepth showed particularly low performance in any mocks even though ANI is systematically different, suggesting that the usage of a reference genome does not appear to affect the precision significantly in our study.

There are still several remaining tasks that we intend to address. First, the relatively low Spearman’s correlation coefficients (< 0.5) suggest that challenges remain in abundance estimation. The vast majority of species occupy less than 5% of each mock, likely making it difficult to precisely determine the abundance ranks of rare species, even with shotgun sequencing. Higher sequencing coverage is expected to improve precision by reducing stochasticity. The relationship between precision and sequencing depth or taxonomic diversity should be further investigated in future studies. Second, the effect of unequal amounts of data between shotgun sequencing and the *rpoB* metabarcoding should also be investigated in future studies. We performed the *rpoB* metabarcoding under standard sequencing conditions to reflect current practices, potentially introducing greater variability into quantification, although rarefaction curves suggest that this reduced sequencing depth does not affect the observed diversity coverage. Third, the comparison between full-length 16S rRNA gene sequencing and shotgun sequencing should be performed in future studies [46]. Lastly, and importantly, investigating more complex microbial communities will be useful for better reflecting natural microbiota, which can include several hundred taxa in host-associated microbiota, such as those in animals, and several thousand in environmental microbiota, such as soils [47].

Taken together, these results suggest that shotgun sequencing coupled with the Assembly-Binning-Method outperforms *rpoB* metabarcoding. For qualitative taxonomic identification, the Assembly-Binning-Method is robust to gDNA contamination from non-microbial species (such as from *S. frugiperda* in this study) and provides greater resolution. Intriguingly, k-mer methods appear to suffer from false positives in the analyzed mock samples. For quantitative abundance estimation, shotgun sequencing offers more precise results, largely unaffected by the choice of reference genomes or whole-genome amplification. Additionally, the database of reference genome assemblies continues to expand, and the significant reduction in next-generation sequencing costs (approximately €6 per 1Gb throughput using NovaSeq X Plus) has made shotgun sequencing more affordable. The cost of shotgun sequencing is still substantially higher than that of metabarcoding [48, 49]. However, expected reductions in sequencing costs may make shotgun sequencing a more accessible alternative. In situations where sequencing cost is not a limiting factor and samples are expected to be heavily contaminated with host gDNA or contain complex bacterial communities, high-coverage shotgun sequencing can be particularly useful for rapidly saturating rarefaction curves.

One potential limitation of shotgun sequencing is the complexity of bioinformatics pipelines required for its analysis, which could remain a challenging aspect of the methodology. To address this point, computer programming scripts were made available for researchers who want to apply the Assembly-Binning-Method. For researchers who are not familiar with command-line tools, the KBase platform [37] offers a user-friendly interface that allows for the analysis of data using most of the tools described in this publication. Metabarcoding can remain a suitable method for taxonomic assignment, especially with recent advances in long-read sequencing. However, research trends in microbial community studies appear to align with decreasing reliance on specific markers and a shift toward unbiased whole-genome analysis, particularly when accuracy quantification is required.

## Supplementary Information

Supplementary Material 1.

Supplementary Material 2

## Data Availability

The sequencing reads generated in this study are available at the NCBI (PRJEB81812 for *rpoB* metabarcoding and PRJEB86763 for shotgun sequencing). Computer programming scripts used in this study are available at https://github.com/karinedurand/Metagenomic/.

## Abbreviations

ANI: Average nucleotide identity
ASV: Amplicon Sequence Variants
CovDepth: Coverage Depth
CovDepth_MAG: Coverage Depth of Metagenome-Assembled Genomes
CovDepth_NCBI: Coverage Depth of NCBI reference genomes
CovDepth_Skani: Coverage Depth from Genome Taxonomy Database using skani
DNA: Deoxyribonucleic Acid
FROGS: Find, Rapidly, OTUs with Galaxy Solution
gDNA: Genomic DNA
GTDB: Genome Taxonomy Database
MAGs: Metagenome-assembled genomes
NCBI: National Center for Biotechnology Information
PCR: Polymerase Chain Reaction
*rpoB*: RNA polymerase beta-subunit gene
rRNA: Ribosomal RNA
