# Supplementary material for: The evaluation of shotgun sequencing and rpoB metabarcoding for taxonomic profiling of bacterial communities
Source: BMC Microbiol. 2025 Jul 4;25:413. doi: 10.1186/s12866-025-04149-3 (PMC12232104; doi:10.1186/s12866-025-04149-3)
Supplement: Supplementary file 2 — Supplementary Material 2 [file 12866_2025_4149_MOESM2_ESM.docx]

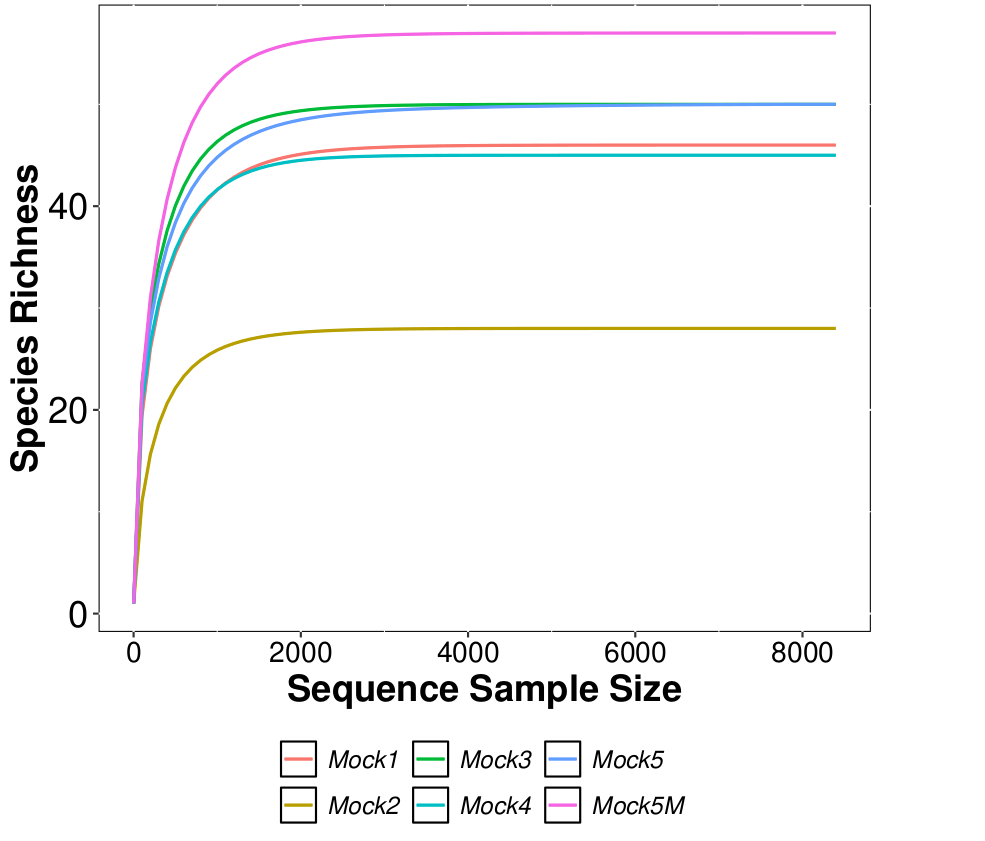


Figure S1 : Rarefaction curves for the rpoB metabarcoding sequences. For each sample, species richness is displayed on the y-axis and the number of sequences on the x-axis. The Rarefy_even_depth function normalizes the samples to the same sequencing depth; the ggrare function was used to generate the rarefaction curves.


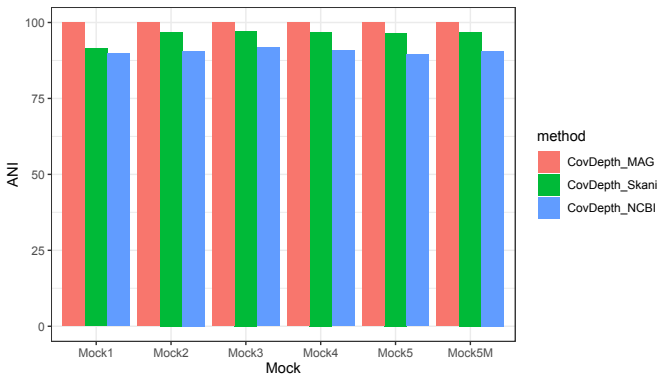
Figure S2. The calculated ANI for each Mock for each CovDepth Method.


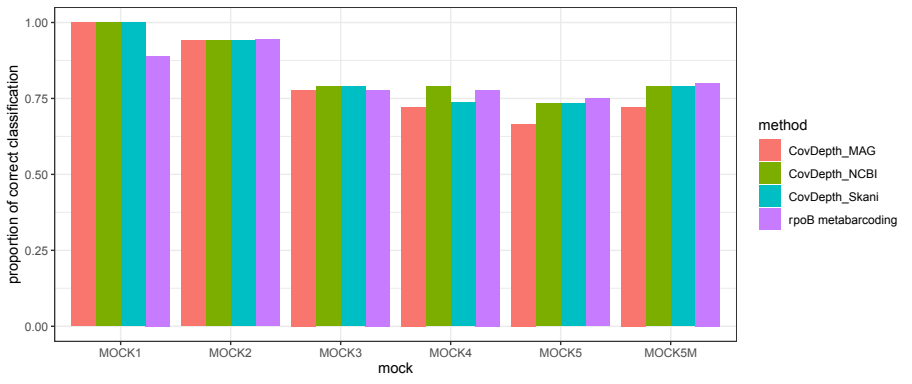
Figure S3. The proportion of correct classifications into abundant (≥10%) or rare (<10%) taxa for each mock and each method
